# Treatment of ovarian endometriomas using plasma energy in endometriosis surgery: effect on pelvic pain, return to work, pregnancy and cyst recurrence

**Published:** 2019-03

**Authors:** EK Lockyer*, AMF Schreurs*, MCI Lier, JJML Dekker, I Melgers, V Mijatovic

**Affiliations:** Amsterdam University Medical Centre, location VUmc, Endometriosis Centre, Department of Reproductive Medicine, De Boelelaan 1118, PK5X194, 1081HV Amsterdam, the Netherlands.

**Keywords:** Endometrioma, plasma energy ablation, fertility, recurrence, recovery

## Abstract

**Background:**

The best surgical technique for managing ovarian endometriomas is still widely debated, though the current standard is stripping cystectomy. The use of plasma energy as a treatment option is a relatively new concept and little data is currently available on this method. The aim of this study was to determine the feasibility of the use of plasma energy in our daily clinical practice by looking at various postoperative outcomes.

**Methods:**

Twenty-one women previously diagnosed with uni- or bilateral ovarian endometriomas by transvaginal ultrasound, associated with pelvic pain and/or infertility, were included in this retrospective cohort study performed in a tertiary endometriosis referral centre. All women underwent endometriotic cyst ablation using plasma energy. At follow up postoperative pain, number of days until return to work following surgery, postoperative pregnancy rate and recurrence rate were determined.

**Results:**

This study demonstrates a significant decrease in the proportion of patients reporting pain postoperatively when comparing the number of patients with dysmenorrhoea, dyspareunia, and chronic pelvic pain pre- and postoperatively. In addition, the median number of days until women returned to work postoperatively was 9 days (interquartile range (IQR) 8-11 days). The postoperative pregnancy rate was 46.2% (6 of 13 women wishing to conceive) and the recurrence rate was 9.5%.

**Conclusions:**

In conclusion, plasma energy is a promising alternative to stripping cystectomy, as comparable results for postoperative pregnancy and recurrence rates can be observed. However, further research is necessary to draw firm conclusions when comparing these two techniques.

## Introduction

Endometriomas are ovarian cysts formed by endometriotic deposits within the ovary, and occur in 17% to 44% of women with endometriosis (Busacca and Vignali, 2003). The primary indications for surgical treatment in these patients are pelvic pain and/or infertility (Vercellini, 1997).

The biggest challenge in operative management of endometriomas is to ensure complete cyst removal and reducing the risk of recurrence whilst maintaining ovarian reserve, as many of the affected women are of childbearing age.

Stripping cystectomy is considered the standard recommended treatment since a Cochrane review published by Hart et al. (2008). However, increasing evidence exists about the negative effect of stripping cystectomy on ovarian reserve through inadvertent removal of ovarian parenchyma along with the endometrioma wall (Hachisuga et al., 2002; Somigliana et al., 2003; Muzii et al., 2005; Ragni et al., 2005; Matsuzaki et al., 2009; Roman et al., 2010; Almog et al., 2011; Hirokawa et al., 2011; Raffi et al., 2012).

A new technique currently being trialled is ablation using plasma energy. Limited data is available on this technique and a randomized controlled trial comparing stripping cystectomy and ablation using plasma energy is yet to be published. The data that is available from a series of non-comparative case-control studies report encouraging results and suggest that ovarian endometrioma ablation using plasma energy is a promising alternative to stripping cystectomy (Auber et al., 2011; Roman et al., 2011a; Roman et al., 2013; Mircea et al., 2016; Motte et al., 2016). The aim of our pilot study was to determine if similar results for recurrence, pregnancy rate, postoperative pain and return to work following surgery could be reproduced when using a plasma energy device in our centre.

## Materials and methods

### Ethical approval

Institutional review board approval was not required for this retrospective study.

### Patients

We performed a retrospective cohort study of all the women who underwent unilateral or bilateral ovarian endometrioma ablation using plasma energy (PlasmaJet® system; Plasma Surgical, Inc., Roswell, GA) between February 2015 and February 2016 at the VU University Medical Centre (VUmc), Amsterdam, the Netherlands. The VUmc is a tertiary endometriosis referral centre. During the study period 65 patients underwent laparoscopic surgery for treatment of endometriotic cysts at the VUmc. All 65 patients were informed about the use of plasma energy and 21 patients elected for ablation using plasma energy.

### Preoperative examination

Preoperatively patients underwent transvaginal ultrasound examination to confirm the presence and record the dimensions of the endometriomas.

### Surgery

The surgical procedures were performed by three gynaecologists (IM, JD, VM), all of whom are specialists in endometriosis and reproductive medicine and are trained and certified in the use of plasma energy (in pigs and humans). All three gynaecologists are also experienced in the use of CO2 lasers for endometriosis surgery. 

Ovarian endometrioma ablation was performed as previously described by Roman et al. (2011a,2011b; 2013). During each procedure a biopsy was taken for histological diagnosis of ovarian endometriosis. Additional procedures were subsequently performed using the plasma energy device if necessary, including vaporization of superficial peritoneal lesions, adhesiolysis, salpingectomy, oophorectomy and rectal shaving, with the aim of achieving complete surgical treatment of all lesions.

### Postoperative examination

Pain scores were recorded preoperatively at the outpatient clinic, post-operatively prior to discharge and at follow up 6-8 weeks later in the outpatient clinic by the attending gynaecologist using the Visual Analogue Scale (VAS) in all cases. The Endometriosis Fertility Index (EFI) was calculated for all patients. If their chances of spontaneous conception in the next 12 months were lower than 30%, which corresponds to an EFI score of ≤ 6, they were referred for medically assisted reproduction (MAR) postoperatively.

The most recent postoperative pelvic ultrasound examination results were collected and used to determine the endometrioma recurrence rate.

The electronic medical records were individually reviewed and information was sourced from pre- and postoperative outpatient clinic records, as well as intraoperative records and inpatient notes. The primary outcomes of this study were incidence and severity of pain postoperatively. Secondary outcomes measured were recurrence of ovarian endometriomas, pregnancy following surgery, and return to work. Recurrence was defined as the presence of a homogenous hypo-echogenic cyst on the ablated ovary on the most recent postoperative transvaginal ultrasound examination. Pregnancy was diagnosed by serum Beta human chorionic gonadotropin and confirmed by the presence of a gestational sac seen on transvaginal ultrasound examination performed at 8 weeks gestation. Return to work was considered the number of days following surgery until women returned to work.

The data collected included demographic data, incidence and severity of pain pre- and postoperatively, use of hormone therapy, subfertility and MAR pre- and postoperatively, intraoperative findings and perioperative characteristics, complications and postoperative outcomes (recurrence, pregnancy, and return to work). Women were considered subfertile if attempts to conceive were unsuccessful for longer than 12 months.

Follow up was variable and was dependent on timing and frequency of outpatient clinic attendance postoperatively. The most recent postoperative outpatient clinic visit before we concluded data collection in December 2017 was considered the end of follow up.

### Data analysis

Statistical analysis was performed using the Statistical Package for the Social Sciences version 22.0 (IBM Corp., Armonk, NY, USA). Categorical data were reported as absolute numbers and percentages. Normally distributed continuous variables were reported as a mean with standard deviation, and non-normally distributed continuous variables were reported as a median with a minimum-maximum range or with interquartile ranges. Continuous outcomes were analysed using an independent T-test or Mann-Whitney U-test as appropriate. We analysed the effects of blood loss, duration of surgery, and size of ovarian endometrioma on return to work with Spearman’s rank-order correlations. The cut-off value for return to work was based on the median number of days.The differences in proportion of patients (pre- and postperatively) with dysmenorrhea, dyspareunia, and for chronic pelvic pain were tested using the McNemar test for paired dichotomous data. A P-value of < 0.05 was considered to be statistically significant.

## Results

From February 2015 to February 2016, 21 women underwent ablation of ovarian endometriomas using plasma energy. All the included women had at least one ovarian endometrioma with a diameter of 25 mm or more associated with pain and/or subfertility. Four patients did not report any pain prior to surgery, however all four were subfertile. The diameters of these patients’ cysts were 25 mm, 30 mm, 30 mm and 40 mm.

Patient demographic data and baseline clinical characteristics are shown in Table I, including fertility history and endometriosis related pain symptoms. In all the patients receiving hormone therapy preoperatively the pain complaints persisted and treatment with hormones did not affect the pain scores. As hormone therapy was not effective for the treatment of pelvic pain in those patients, they were referred for surgical treatment.

**Table I:** — Patient characteristics and obstetric & gynaecologic history.

			N=21	
Patient characteristic				
	Age (years)		31.8	± 5.9
	BMI		23.9	± 4.1
Obstetric history				
	Intention to conceive, N (%)		13	(61.9)
	Subfertile (of those intending to conceive), N (%)		12	(92.3)
	Duration subfertility (months)		34	± 16.4
	Previous MAR management, N (%)		6	(28.6)
		IVF	1	(4.8)
		ICSI	2	(9.5)
		Insemination	4	(19)
Gynaecologic history				
	Preoperative hormone therapy, N (%)		10	(47.6)
		GnRH analogues, N (%)	2	(9.5)
		Oral contraceptive pill, N (%)	7	(33.3)
		Progestins, N (%)	1	(4.8)
		Duration of hormone therapy (months)	6.5	± 4.0
	Symptoms related to endometriosis			
		Chronic pelvic pain, N (%)	12	(57.1)
		VAS score chronic pelvic pain (0-10 cm)	5	[0-8]
		Dysmenorrhoea, N (%)	16	(76.2)
		VAS score dysmenorrhoea (0-10 cm)	6	[0-8]
		Dyspareunia, N (%)	8	(38.1)
		VAS score dyspareunia (0-10 cm)	0	[0-7]
	Endometrioma characteristics			
		Unilateral, N (%)	19	(90.5)
		Bilateral, N (%)	2	(9.5)
		Size (mm)	41.7	± 12.1

All data are means ±SD or medians with minimum-maximum ranges

Abbreviations: BMI, body mass index; ICSI, intracytoplasmic sperm injection; IVF, in vitro fertilization; MAR, medically assisted reproduction; VAS, visual analogue scale

Table II lists the intraoperative findings and surgical procedures performed. All procedures were performed laparoscopically and none of the women required conversion to laparotomy or experienced any intraoperative or postoperative complications.

**Table II:** — Intraoperative findings and surgical procedures performed.

			N=21	
Operative time (min)			90.6	± 27
Duration of ovarian endometrioma vaporization (min)			16.7	± 4.1
Inversion of inner cyst wall, N (%)			8	(38.1)
Bipolar coagulation required, N (%)			6	(28.6)
Total blood loss (mL)			60	[<50-300]
Cyst wall pathology results from intraoperative biopsy, N (%)				
	Confirmed endometriosis		16	(76.2)
	Probable endometriosis		3	(14.3)
	Inconclusive		2	(9.5)
Additional procedures performed, N (%)				
	Adhesiolysis		21	(100)
		Right adnexa	11	(52.4)
		Left adnexa	14	(66.7)
		Peritoneum	18	(85.7)
		Omentum	1	(4.8)
		Rectosigmoid	1	(4.8)
	[Rectal] shaving		2	(9.5)
	Salpingectomy		2	(9.5)
	Salpingectomy and oophorectomy		1	(4.8)

All data are means ±SD or medians with minimum-maximum ranges.

Abbreviations: GnRH, gonadotropin-releasing hormone.

The follow up time of our study was relatively varied, with a median follow up time of 10 months, and ranging from 3 to 31 months.

Postoperative outcomes are summarized in Table III. All 13 women who wished to conceive were referred for MAR postoperatively within 3 months. Following surgery the EFI was calculated, and patients with a score of < 3 were referred for in vitro fertilisation (IVF). Patients with an EFI score between 4 and 6 were referred for intrauterine insemination (IUI). Six of these women fell pregnant following MAR and gave birth within our follow up period, 3 of them by caesarean section, and 3 by vaginal delivery. No miscarriages occurred during the follow up period. Endometrioma recurrence was established in 2 women (9.5%). One other patient had a visible ovarian endometrioma on ultrasound postoperatively, however because the cyst was not on the operated side this was not considered a recurrence.

**Table III:** — Postoperative outcomes.

			N=21	
Reproductive outcomes				
Women with intention to conceive in follow-up period, N (%)			13	(61.9)
	Postoperative MAR management, N (%)		13	(100)
		IVF	7	(53.8)
		ICSI	6	(46.2)
		Insemination	7	(53.8)
		Transfer of frozen embryos (prior IVF)	1	(7.7)
	Pregnancy, N (%)		6	(46.2)
	Method of conception (N=6), N (%)			
		IVF	3	(60)
		ICSI	1	(20)
		Insemination	2	(20)
	Live births, N (%)		6	(46.2)
Other postoperative outcomes				
Recurrence of endometriosis pain symptoms				
	Chronic pelvic pain, N (%)		3	(14)
	VAS scores chronic pelvic pain (0-10 cm)		0	[0-6]
	Dysmenorrhoea, N (%)		4	(19)
	VAS score dysmenorrhoea (0-10 cm)		0	[0-4]
	Deep dyspareunia, N (%)		1	(4.8)
	VAS score dyspareunia (0-10 cm)		0	[0-3]
	VAS score for postoperative pain on discharge (0-10 cm)		1.6	± 0.9
	Recurrence of endometrioma, N (%)		2	(9.5)
	Days until return to work postoperatively (days)		9.0	(IQR 8-11)
Postoperative hormone therapy, N (%)			14	(66.7)
	None		7	(33.3)
	Oral contraceptive pill		5	(23.8)
	GnRH		8	(38.1)
	Progestins		1	(4.8)

All data are means ±SD or medians with minimum-maximum ranges or with interquartile ranges (IQR)

Abbreviations: GnRH, gonadotropin releasing hormone; ICSI, intracytoplasmic sperm injection; IVF, in vitro fertilization; MAR, medically assisted reproduction; VAS, visual analogue scale.

**Figure 1:**
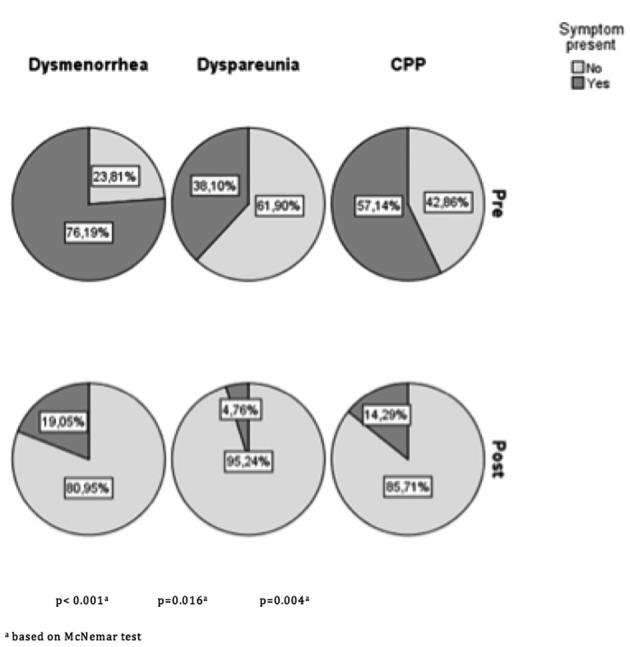
Proportions of patients with pre- and postopertive pain complaints.

There was a statistically significant decrease in the proportion of patients reporting dysmenorrhoea, dyspareunia, and chronic pelvic pain postoperatively, as well as a considerable reduction in the pain scores when comparing women pre-and postoperatively. This is illustrated in Figure 1 and Table III. The median number of days until women returned to work following surgery was 9 days (IQR 8-11 days). Finally, as shown in Table IV, we observed that larger volumes of blood loss, a longer duration of the surgery, and increased size of ovarian endometrioma led to a longer duration until return to work, and that these relationships were statistically significant.

**Table IV:** — Effect of blood loss, duration of surgery, and size of ovarian endometrioma on return to work.

	Days until return to work ≤ 9	Days until return to work > 9	p-value*
Total blood loss (mL)	<50 (<50-62,5)	150 (80-300)	< 0.001
Operative time (min)	80 (59,3-90)	125 (110-130)	< 0.001
Size of ovarian endometrioma (mm)	40 (30-42,5)	55 (40-70)	0.03

All data are medians with interquartile ranges.

*Mann-Whitney U test

## Discussion

This retrospective cohort study revealed similar intra- and postoperative outcomes to those previously reported. In addition we observed a significant decrease in post-operative pain scores and a quick postoperative recovery, suggesting that ablation using plasma energy might be a promising new surgical technique for the treatment of ovarian endometriomas. Extensive research has been done regarding the most suitable surgical technique for the management of ovarian endometriomas and many different approaches have been trialled, the most common of which being (laparoscopic) stripping cystectomy; ablation, including electrical/thermal and CO2 laser ablation; fenestration/aspiration; and combined techniques (Jadoul et al., 2012). The use of plasma energy for the management of ovarian endometriomas is a fairly new concept, having only been implemented since 2009 (Mircea et al., 2016; Ragni et al., 2005). A plasma energy device is similar to a CO2 laser in that it destroys the tissue without coagulum disruption. It does this using argon gas and is reported to have no risk of accidental intraoperative overshoots or metallic instrument reflection (Nezhat et al., 2009; Deb et al., 2010).

One of our principal objectives was to look at the efficacy of this new technique for pain reduction postoperatively. We observed a considerable reduction in the VAS scores postoperatively as well as a statistically significant decrease in the proportion of patients with dysmenorrhoea, dyspareunia and chronic pelvic pain postoperatively. A statistically significant decrease in dysmenorrhoea VAS scores was also found by Roman et al. (2014), though this study as well as the study by Mircea et al. (2016) did not report statistically significant differences in pre- and postoperative VAS scores for dyspareunia or chronic pelvic pain (Mircea et al., 2016; Roman et al., 2014). It should be noted however that the [necessity for] additional procedures performed in some cases are also likely to have affected pre- and postoperative pain scores. We found our results for return to work to be equally positive, as the median number of days until patients returned to work postoperatively was 9.0 days (IQR 8-11). This is a novel finding, as this postoperative outcome has not been reported in other studies using plasma energy to treat endometriosis. However, when compared to results published by Vonk Noordegraaf et al. (2014) on return to work after benign gynaecological surgery, our patients had a considerably shorter recovery period to patients undergoing similar laparoscopic gynaecological operations, for which the median duration of return to work was 14 days. As expected, subgroup analysis revealed that blood loss, duration of surgery, and size of ovarian endometrioma independently influenced the duration until return to work.

According to Roman et al. (2011a) the duration of ovarian endometrioma vaporization is usually 10 to 20 minutes. Our results for mean vaporization time are similar to those demonstrated by Roman et al. (2014), averaging at 16.7 minutes. The average duration of surgery was 90.6 minutes for the women operated on in our hospital, however as varying additional procedures were performed in the majority of cases, operative time cannot be compared to other studies where the use of plasma energy was employed. Similarly, results for intraoperative blood loss should not be compared.

Recent data published in a number of case series has demonstrated that the rates of recurrence and postoperative pregnancy in women following ovarian endometrioma ablation using a plasma energy device are comparable to the rates previously reported in women in whom other surgical approaches were employed (Donnez et al., 1996; Beretta et al., 1998; Alborzi et al., 2004; Tsolakidis et al., 2010; Carmona et al., 2011; Roman et al., 2013;2015; Mircea et al., 2016; Motte et al., 2016).

The pregnancy rate in our retrospective study was 46.2%, as 6 of the 13 women wishing to conceive fell pregnant and delivered within the follow-up period. Though our postoperative pregnancy rates are lower than those seen in women from the CIRENDO (the North-West Inter Regional Female Cohort for Patients with Endometriosis) database in France (ranging from 56.8%-68.7%) (Donnez et al., 1996; Roman et al., 2013;2015; Mircea et al., 2016; Motte et al., 2016), our results are still comparable to pregnancy rates reported following stripping cystectomy which range from 30 to 67% (Fedele et al., 2006; Vercellini et al., 2009; Carmona et al., 2011; Berlanda et al., 2013). However, it should be noted that in our study the pregnancies were achieved by MAR. Previous studies using plasma energy to manage ovarian endometriomas described having recurrence rates ranging from 5 to 14.5% (Roman et al., 2013;2014;2015); which are comparable to those following stripping cystectomy, which range from 6.2 to 29% (Beretta et al., 1998; Alborzi et al., 2004; Sesti et al., 2009; Seracchioli et al., 2010; Carmona et al., 2011). Our findings corroborate such results as we demonstrated a recurrence rate of 9.5% following the use of a plasma energy device. Our follow-up time, the median being 10 months, is however shorter than the average follow-up time observed in the studies by Roman et al. (2013; 2015), Mircea et al. (2016) and Motte et al. (2016), ranging from 20.6 to 36 months. A larger cyst diameter is usually considered a risk factor for recurrence. This is corroborated by our results as well, as the two patients with cyst recurrence had pre-operative cyst diameters of 50mm and 60mm, which is larger than our average cyst size of 41.7mm. The average cyst diameter in our study group is however comparable to the average cyst diameters described in the studies by Roman et al. (2013; 2014; 2015) and Motte et al. (2016).

The principal limitations of our study are comprised of the inherent methodological limitations that tend to accompany a retrospective pilot study lacking a control group such as this one. In addition, the variable follow-up time, owing to varying clinical factors and resulting differences in necessary management, is a disadvantage and makes comparison to other studies difficult. We can therefore not say with certainty whether the use of plasma energy alone may account for the results presented in this study or whether the results were the consequence of, or influenced by, the (pre-) operative workup.

Conversely, a major strength of our study is related to our investigating in more detail the efficacy of this technique in terms of improvement in pain following surgery, in addition to pregnancy and recurrence rates. It is necessary to consider these factors as well in order to determine how well a plasma energy device can be implemented in our daily practice and whether our patient population can benefit. Whilst it would be ideal to observe this in a larger trial in the future, our findings for pain scores postoperatively presented in this study are promising.

## Conclusion

Our results suggest that the use of plasma energy for the management of ovarian endometriomas is a feasible and an attractive alternative to stripping cystectomy.

Nevertheless, no definitive conclusions can be drawn until randomized trials are performed comparing plasma energy ablation to other management approaches in the treatment of endometriomas, including stripping cystectomy. 

## References

[1] Alborzi S, Momtahan M, Parsanezhad ME (2004). A prospective, randomized study comparing laparoscopic ovarian cystectomy versus fenestration and coagulation in patients with endometriomas.. Fertil Steril.

[2] Almog B, Shehata F, Sheizaf B (2011). Effects of ovarian endometrioma on the number of oocytes retrieved for in vitro fertilization.. Fertil Steril.

[3] Auber M, Bourdel N, Mokdad C (2011). Ultrasound ovarian assessments after endometrioma ablation using plasma energy. Fertil Steril.

[4] Beretta P, Franchi M, Ghezzi F (1998). Randomized clinical trial of two laparoscopic treatments of endometriomas: cystectomy versus drainage and coagulation.. Fertil Steril.

[5] Berlanda N, Vercellini P, Somigliana E (2013). Role of surgery in endometriosis-associated subfertility.. Semin Reprod Med.

[6] Busacca M, Vignali M (2003). Ovarian endometriosis: from pathogenesis to surgical treatment.. Curr Opin Obstet Gynecol.

[7] Carmona F, Martinez-Zamora MA, Rabanal A (2011). Ovarian cystectomy versus laser vaporization in the treatment of ovarian endometriomas: a randomized clinical trial with a five-year follow-up.. Fertil Steril.

[8] Deb S, Deen S, Ashford KS (2010). Histological quantification of the tissue damage caused by PlasmaJet Coagulator.. Gynecol Surg.

[9] Donnez J, Nisolle M, Gillet N (1996). Large ovarian endometriomas.. Hum Reprod.

[10] Fedele L, Bianchi S, Zanconato G (2006). Laparoscopic excision of recurrent endometriomas: long-term outcome and comparison with primary surgery.. Fertil Steril.

[11] Hachisuga T, Kawarabayashi T (2002). Histopathological analysis of laparoscopically treated ovarian endometriotic cysts with special reference to loss of follicles.. Hum Reprod.

[12] Hart RJ, Hickey M, Maouris P (2008). Excisional surgery versus ablative surgery for ovarian endometrioma.. Cochrane Database Syst Rev.

[13] Hirokawa W, Iwase A, Goto M (2011). The post-operative decline in serum anti-Mullerian hormone correlates with the bilaterality and severity of endometriosis.. Hum Reprod.

[14] Jadoul P, Kitajima M, Donnez O (2012). Fertil Steril. Surgical treatment of ovarian endometriomas: state of the art.

[15] Matsuzaki S, Houlle C, Darcha S (2009). Analysis of risk factors for the removal of normal ovarian tissue during laparoscopic cystectomy for ovarian endometriosis.. Hum Reprod.

[16] Mircea O, Puscasiu L, Resch B (2016). Fertility outcomes after ablation using plasma energy versus cystectomy in infertile women with ovarian endometrioma: a multicentric comparative study.. J Minim Invasive Gynecol.

[17] Motte I, Roman H, Clavier B (2016). In vitro fertilization outcomes after ablation of endometriomas using plasma energy: a retrospective case-control study.. Gynecologie Obstetrique & Fertilite.

[18] Muzii L, Bellati F, Bianchi A (2005). Laparoscopic stripping of endometriomas: a randomized trial on different surgical techniques. Part I: pathological results. Hum Reprod.

[19] Nezhat C, Kho KA, Morozov V (2009). Use of neutral argon plasma in the laparoscopic treatment of endometriosis.. JSJS.

[20] Raffi F, Metwally M, Amer S (2012). The impact of excision of ovarian endometrioma on ovarian reserve: A systematic review and meta-analysis. J Clin Endocrinol Metab.

[21] Ragni G, Somigliana E, Benedetti F (2005). Damage to ovarian reserve associated with laparoscopic excision of endometriomas: a quantitative rather than a qualitative injury.. Am J Obstet Gynecol.

[22] Roman H, Auber M, Bourdel N (2013). Postoperative recurrence and fertility after endometrioma ablation using plasma energy: retrospective assessment of a 3 year experience.. J Minim Invasive Gynecol.

[23] Roman H, Auber M, Mokdad C (2011a). Ovarian endometrioma ablation using plasma energy versus cystectomy: a step toward better preservation of the ovarian parenchyma in women wishing to conceive.. Fertil Steril.

[24] Roman H, Bubenheim M, Auber M (2014). Antimullerian hormone level and endometrioma ablation using plasma energy.. JSLS.

[25] Roman H, Pura I, Tarta O (2011b). Vaporization of ovarian endometrioma using plasma energy: histological findings of a pilot study.. Fertil Steril.

[26] Roman H, Quibel S, Auber M (2015). Recurrences and fertility after endometrioma ablation in women with and without colorectal endometriosis: a prospective cohort study.. Hum Reprod.

[27] Roman H, Tarta O, Pura I (2010). Direct proportional relationship between endometrioma size and ovarian parenchyma inadvertently removed during cystectomy, and implication on the management of enlarged endometriomas.. Hum Reprod.

[28] Seracchioli R, Mabrouk M, Frasca C (2010). Long-term cyclic and continuous oral contraceptive therapy and endometrioma recurrence: a randomized controlled trial.. Fertil Steril.

[29] Sesti F, Capozzolo T, Pietropolli A (2009). Recurrence rate of endometrioma after laparoscopic cystectomy: a comparative randomized trial between post-operative hormonal suppression treatment or dietary therapy vs. placebo. Eur J Obstet Gynecol Reprod Biol.

[30] Somigliana E, Ragni G, Benedetti F (2003). Does laparoscopic excision of endometriotic ovarian cysts significantly affect ovarian reserve? Insights from IVF cycles.. Hum Reprod.

[31] Tsolakidis D, Pados G, Vavilis D (2010). The impact of ovarian reserve after laparoscopic ovarian cystectomy versus three-stage management in patients with endometriomas: a prospective randomized study.. Fertil Steril.

[32] Vercellini P, Somigliana E, Vigano P (2009). Surgery for endometriosis-associated infertility: a pragmatic approach.. Hum Reprod.

[33] Vercellini P (1997). Endometriosis: what pain it is.. Seminars in Reproductive Endocrinology.

[34] Vonk Noordegraaf A, Anema JR, Louwerse MD (2014). Prediction of time to return to work after gynaecological surgery: a prospective cohort study in the Netherlands.. BJOG.

